# Mutagenesis of the Loop 3 α-Helix of Neisseria gonorrhoeae TdfJ Inhibits S100A7 Binding and Utilization

**DOI:** 10.1128/mbio.01670-22

**Published:** 2022-07-14

**Authors:** Stavros A. Maurakis, Julie L. Stoudenmire, Jeffrey K. Rymer, Walter J. Chazin, Cynthia Nau Cornelissen

**Affiliations:** a Institute for Biomedical Sciences, Georgia State University, Atlanta, Georgia, USA; b Department of Biochemistry, Vanderbilt University, Nashville, Tennessee, USA; c Department of Chemistry, Vanderbilt University, Nashville, Tennessee, USA; Emory University School of Medicine

**Keywords:** *Neisseria gonorrhoeae*, nutritional immunity, S100A7, TonB-dependent transporter

## Abstract

Neisseria gonorrhoeae causes the sexually transmitted infection (STI) gonorrhea, which afflicts over 80 million people each year. No vaccine is available to prevent gonorrhea. The pathogen alters the expression and antigenic presentation of key surface molecules, making the identification of suitable vaccine targets difficult. The human host utilizes metal-binding proteins to limit free essential transition metal ions available to invading pathogens, limiting their infective potential, a process called nutritional immunity. To overcome this, N. gonorrhoeae employs outer membrane TonB-dependent transporters (TdTs) that bind host nutritional immunity proteins and strip them of their metal cargo. The TdTs are well conserved, and some play key roles in establishing infections, making them promising vaccine targets. One TdT, TdfJ, recognizes human S100A7, a zinc-binding protein that inhibits the proliferation of other pathogens via zinc sequestration. N. gonorrhoeae uses TdfJ to strip and internalize zinc from S100A7. TdfJ contains a conserved α-helix finger in extracellular loop 3; a similar α-helix in loop 3 of another gonococcal TdT, TbpA, plays a critical role in the interaction between TbpA and human transferrin. Therefore, we hypothesized that the TdfJ loop 3 helix (L3H) participates in interactions with S100A7. We determined the affinity between wild-type TdfJ and S100A7 and then generated a series of mutations in the TdfJ L3H. Our study revealed that mutagenesis of key residues within the L3H reduced S100A7 binding and zinc piracy by the gonococcus, with profound effects seen with substitutions at residues K261 and R262. Taken together, these data suggest a key role for the TdfJ L3H in subverting host metal restriction.

## INTRODUCTION

Neisseria gonorrhoeae causes the common sexually transmitted infection (STI) gonorrhea, a global disease with serious public health consequences. Gonorrhea infected an estimated 87 million people worldwide in 2016, and the Centers for Disease Control and Prevention (CDC) reported 616,392 cases of gonorrhea in the United States in 2019 (1). Medical costs associated with gonococcal disease in the United States have reached $271 million (2). Gonorrhea infects both men and women. In men, primary infections present as epididymitis and urethritis, and in women, they present as cervicitis, although up to 80% of cases in women are asymptomatic (3). If left untreated, gonorrhea can lead to serious secondary sequelae, including pelvic inflammatory disease, ectopic pregnancy, infertility, and even life-threatening endocarditis and meningitis (4–6).

Effective treatment options for gonorrhea are decreasing, as N. gonorrhoeae naturally acquires and maintains antimicrobial resistance mechanisms with high efficiency. High-frequency resistance to penicillin, sulfonamides, and quinolones is already present, and extended-spectrum cephalosporins have shown treatment failure (7–10). Until recently, the CDC-recommended therapy for gonococcal infection was dual treatment with ceftriaxone and azithromycin, but azithromycin has been dropped from this regimen (11), and the incidence of ceftriaxone resistance continues to increase (12). In addition, no effective vaccine to prevent gonorrhea has been identified to date (for a review, see reference 13), and a protective immune response is not conferred upon natural infection (14). Taken together, these facts highlight the threat that gonorrhea may become untreatable and that novel therapeutic and/or preventative measures are needed.

A promising set of targets for such a vaccine or therapeutic is the outer membrane TonB-dependent transporters (TdTs). The TdTs are highly conserved across gonococcal isolates and play key roles in overcoming nutritional immunity, the process by which the human host restricts essential nutrients to handicap invading pathogens (reviewed in references 15–17). Recently, we showed that one TdT, TdfJ, binds the human innate immunity protein S100A7 and enables zinc extraction by the gonococcus (18). This interaction is the first of its kind reported for S100A7, which typically exhibits an inhibitory effect on bacterial growth by virtue of its zinc sequestration capabilities (19–23) and in some cases even shows contact-dependent killing of microbes (24).

The Neisseria meningitidis homologue of TdfJ, called ZnuD, is nearly identical (>97%) to TdfJ in amino acid sequence. The crystal structure of ZnuD (PDB accession numbers 4RDR and 4RDT) shows that extracellular loop 3 contains two zinc-sensing regions, which are enriched in histidine, aspartate, and glutamate. Furthermore, this region undergoes considerable rearrangement in a substrate-dependent manner. When zinc is absent, the region of loop 3 between the zinc-sensing clusters adopts an α-helical configuration that extends far from the TdT barrel. Conversely, in the presence of zinc, this region is remodeled into a pair of flexible β-strands, exposing zinc in the peripheral binding site to a newly available, high-affinity site buried deeper in the barrel (25). While such a rearrangement has been observed in other zinc-binding proteins (26–28), this is a novel phenomenon among the TdTs. In another study, Cash et al. (29) demonstrated that an α-helix in loop 3 of another TdT, TbpA, plays a vital role in the interaction with, and subsequent iron extraction from, human transferrin. Therefore, we hypothesized that the TdfJ loop 3 helix (L3H) plays a similar role in the binding of, and zinc extraction from, S100A7. In this report, we determined the affinity of wild-type (WT) TdfJ and S100A7 and then utilized site-directed mutagenesis to identify key amino acid residues of TdfJ involved in binding and subsequent zinc extraction from S100A7.

## RESULTS

### Wild-type TdfJ binds S100A7 with high affinity.

**FIG 1:**
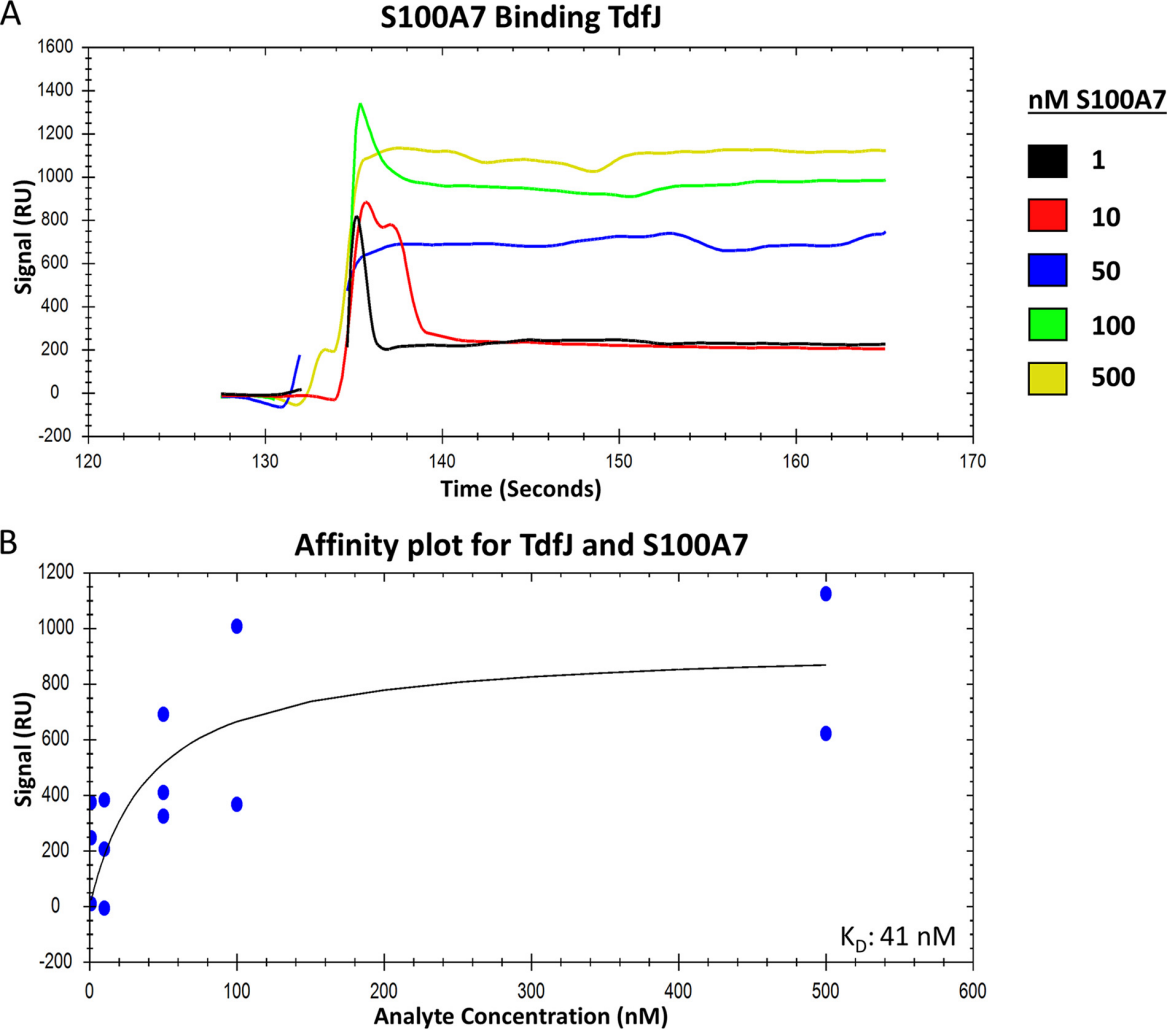
Wild-type TdfJ binds S100A7 with high affinity. His-tagged, wild-type TdfJ was immobilized on an NTA-coated SPR sensor chip, and the remaining free NTA sites were blocked with His-tagged streptavidin. S100A7 was then passed over the TdfJ chip to assess binding. (A) SPR sensorgram showing the baseline, association, and steady-state binding of five concentrations (1, 10, 50, 100, and 500 nM) of S100A7 with wild-type TdfJ. The binding detected is reported as arbitrary response units (RU) on the *y* axis. Gaps in the trace lines represent response spikes, likely generated by air bubbles, which were removed postrun. The plot is a representative trace for 3 experiments. (B) Plot showing the steady-state affinity of S100A7 and TdfJ calculated from multiple analyte injections of each concentration over multiple runs. A line of best fit is shown and was used to calculate a dissociation constant.

We previously reported that whole gonococcal cells presenting TdfJ on their surface can bind S100A7 (18). However, the interaction was not investigated using purified proteins in isolation, suggesting that other membrane factors may play some role in binding. To address this question, we sought to further characterize the interaction between WT TdfJ and S100A7. To this end, we purified WT TdfJ and performed surface plasmon resonance (SPR) to interrogate the binding of S100A7 to TdfJ (Fig. 1A). His-tagged TdfJ was immobilized on a Ni-nitrilotriacetic acid (NTA) sensor chip and subsequently blocked with His-tagged streptavidin. Successive injections of 1, 10, 50, 100, and 500 nM S100A7 were then utilized to characterize the binding interaction. Additions of S100A7 generated a concentration-dependent response ranging from approximately 220 average response units (RU) upon the addition of 1 nM S100A7 to approximately 900 average RU for 500 nM S100A7. Analysis of these sensorgrams revealed a high-affinity interaction between TdfJ and S100A7. A 50% target saturation (ED50) calculation was used to generate a binding curve, and the data were fit to a single-site binding model, yielding a dissociation constant of 41 nM (Fig. 1B). These data corroborate our previous proposal that TdfJ interacts with S100A7 and indicate that the proteins themselves, devoid of any external membrane factors, bind with high affinity. With this in mind, we next sought to mutagenize the putative zinc-sensitive region of TdfJ and assess its impact on S100A7 recognition. We first generated a deletion mutant lacking 15 amino acids from the L3H (ΔL3H) to gauge whether the region in question was a suitable target. Next, with an understanding that such a mutant may have profound impacts on overall protein folding and stability, we focused on point mutations within the original 15-residue segment. These mutations included a proline substitution to physically disrupt the helical motif and charge changes to potentially alter zinc coordination in either TdfJ or S100A7.

### Variant *tdfJ* genes were expressed from an inducible ectopic site in the gonococcal chromosome.

**FIG 2:**
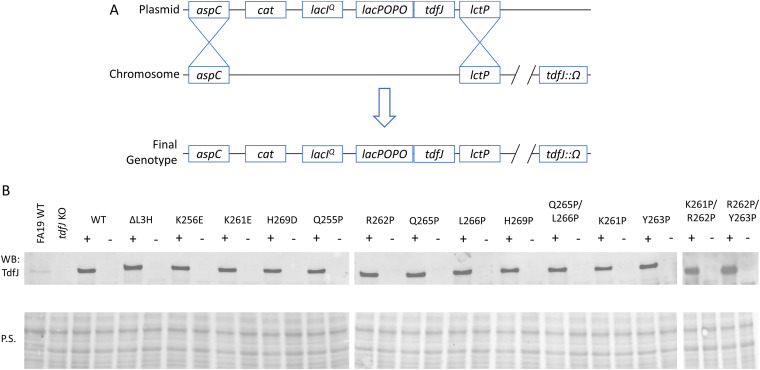
Variant *tdfJ* genes are expressed from an inducible ectopic site in the gonococcal chromosome. Wild-type and mutated forms of *tdfJ* were cloned into a complementation vector designed to insert into an ectopic site of the gonococcal chromosome between the *aspC* and *lctP* loci. This construct contains an IPTG-inducible promoter and a strong ribosome-binding site upstream of the inserted gene. (A) Schematic demonstrating the final genotype of gonococcal *tdfJ* mutants. Plasmids containing either wild-type or mutated *tdfJ* were used to transform N. gonorrhoeae strain FA19 with its native *tdfJ* locus inactivated by an omega cassette, resulting in gonococci that produce only TdfJ under induction. (B) Gonococcal *tdfJ* mutants grown on GCB agar plates with (+) and without (−) 1 mM IPTG were resuspended in PBS. Cell suspensions were standardized and used to prepare cell lysates, which were Western blotted (WB) to assess TdfJ production and the IPTG control. A true wild-type strain, FA19, and its isogenic *tdfJ* mutant were grown under zinc-limited conditions to serve as positive and negative controls. Ponceau staining (P.S.) of the blots is also shown to demonstrate equal loading. (“FA19 WT” refers to the true wild-type strain expressing *tdfJ* from its native locus; “WT” refers to the wild-type *tdfJ* gene in the inducible ectopic site; and “ΔL3H” refers to a deletion mutant in *tdfJ*, which lacks 15 amino acids from the loop 3 α-helix.) The blot is representative of results from 3 experiments.

The native locus of *tdfJ* is maximally expressed only under conditions of low zinc and high iron (18, 30). As such, reliably reproducing equivalent gene expression and protein production profiles for mutated *tdfJ* genes in the native locus promised to be a challenge that may confound comparisons between mutants. As such, we instead chose to perform experiments using the isogenic *tdfJ* mutant strain MCV928 (31), which has an inactivated native locus, and to add back our mutated *tdfJ* genes via the complementation plasmid pVCU234, which contains a strong ribosome-binding site behind a promoter inducible by isopropyl β-d-1-thiogalactopyranoside (IPTG) (32). A schematic of the final genotype is shown in Fig. 2A, and unless otherwise noted, a “WT” strain in this report refers to an unmutated *tdfJ* gene expressed from the complementation locus and not a true WT strain. This method hypothetically allowed more precise control of protein production via the addition of a consistent amount of the inducer and, therefore, more consistent comparisons of protein characteristics.

After generating the inducible mutants, we verified that they produced the correct gene product under IPTG control, as predicted, by performing Western blot analysis of gonococcal *tdfJ* mutants grown with and without IPTG to detect TdfJ production (Fig. 2B). A true WT strain, FA19 (33), was also grown under low-zinc, high-iron conditions to serve as a positive control, and a lysate of strain MCV928 was used as a negative control. The blots showed that each mutant strain overexpressed its respective *tdfJ* gene only when IPTG was present, and no protein was detected when the inducer was absent. With these conditions established, we moved on to further characterization of the mutants.

### TdfJ mutants are surface exposed and mimic the folding of the wild type.

**FIG 3:**
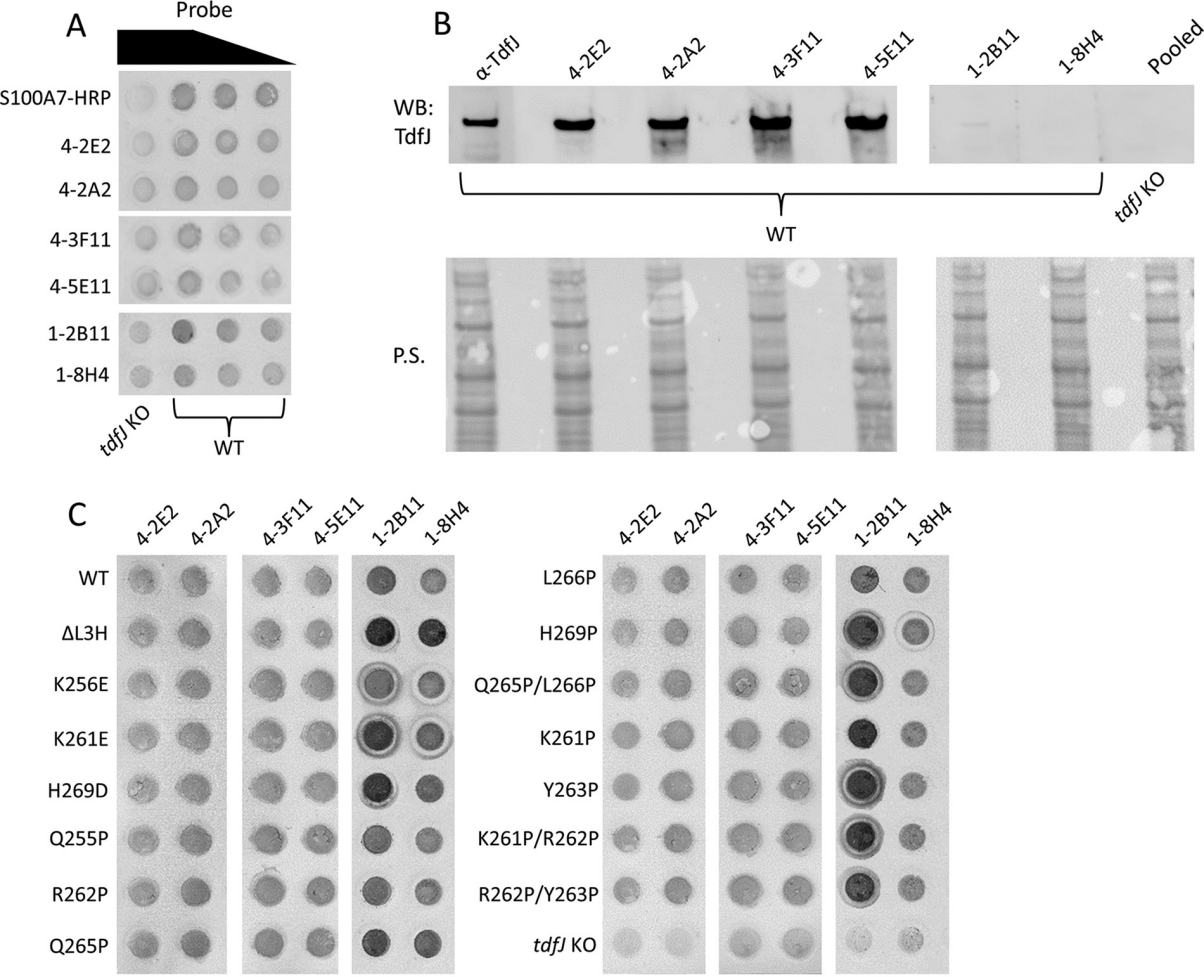
TdfJ mutants are surface exposed and mimic the folding of the wild type. (A) Gonococcal WT and *tdfJ* knockout (*tdfJ* KO) strains were grown on GCB agar plates containing 1 mM IPTG and then resuspended in PBS at an OD_600_ of 1.0. Cell suspensions were dotted onto nitrocellulose in a dot blotter and allowed to adsorb and dry. The dot blot was blocked with BSA and subsequently probed with either HRP-conjugated S100A7 (S100A7-HRP) or TdfJ-specific mouse monoclonal antibodies (mAbs) in a series of dilutions (0.4 → 0.2 → 0.1 → 0.05 μM for S100A7-HRP and 1:10 → 1:50 → 1:100 for mAbs). The blots were then probed with either HRP-conjugated anti-mouse IgG secondary antibodies, and the signal was developed by the addition of the HRP-reactive CN/DAB substrate (for mAbs 4-2E2, 4-2A2, 4-3F11, and 4-5E11), or AP-conjugated anti-mouse IgG secondary antibodies, and the signal was developed by the addition of the AP-reactive NBT-BCIP substrate (for 1-2B11 and 1-8H4). (B) WT and *tdfJ* KO strains were grown as described above, and whole-cell lysates were prepared for SDS-PAGE and Western analysis. Western blots were probed with either anti-TdfJ polyclonal serum (α-TdfJ), one of the six mAbs, or all antibodies pooled for the *tdfJ* KO lysate. Ponceau staining (P.S.) of the blots is shown to demonstrate equal sample loading. (C) Gonococcal *tdfJ* mutants were dotted onto nitrocellulose as described above and, after blocking, probed with each of the six mAbs, which were then detected using the same methods as the ones described above for panel A. The bots shown above are representative of results from 3 experiments.

Before performing S100A7 binding assays, we ensured that the mutations that we introduced into *tdfJ* did not have profound, off-target impacts on TdfJ stability or its presentation on the cell surface, as these would have confounded our analyses. To assess this, we first characterized a panel of TdfJ-specific mouse monoclonal antibodies (mAbs) and identified those that recognized either folded surface-exposed TdfJ, Western-blotted TdfJ, or both (Fig. 3A and B). These validation experiments indicated that only gonococci producing TdfJ, either as whole cells or as lysates, were recognized by the mAbs, as the *tdfJ* knockout (KO) strain was not detected above background levels. The capacity for recognizing TdfJ on the gonococcal cell surface made the mAbs a useful tool for assessing the global fold of mutated forms of TdfJ. We immobilized gonococci producing these mutated proteins on a nitrocellulose membrane and probed their surface with mAbs for comparison to the pattern for the WT (Fig. 3C). We also probed a strain not producing TdfJ to demonstrate specificity. All strains that produced a form of TdfJ were recognized, suggesting that they successfully exported the protein to the surface and that mutation did not affect the TdfJ interaction with the Sec system or the β-barrel assembly machinery (reviewed in reference 34). The mAb binding pattern for WT TdfJ remained consistent across mutant strains and was indistinguishable from that of the WT for most mutants, suggesting that the overall fold and stability of the protein were not perturbed by mutagenesis.

### Mutations in the TdfJ loop 3 α-helix can inhibit S100A7 binding.

**FIG 4:**
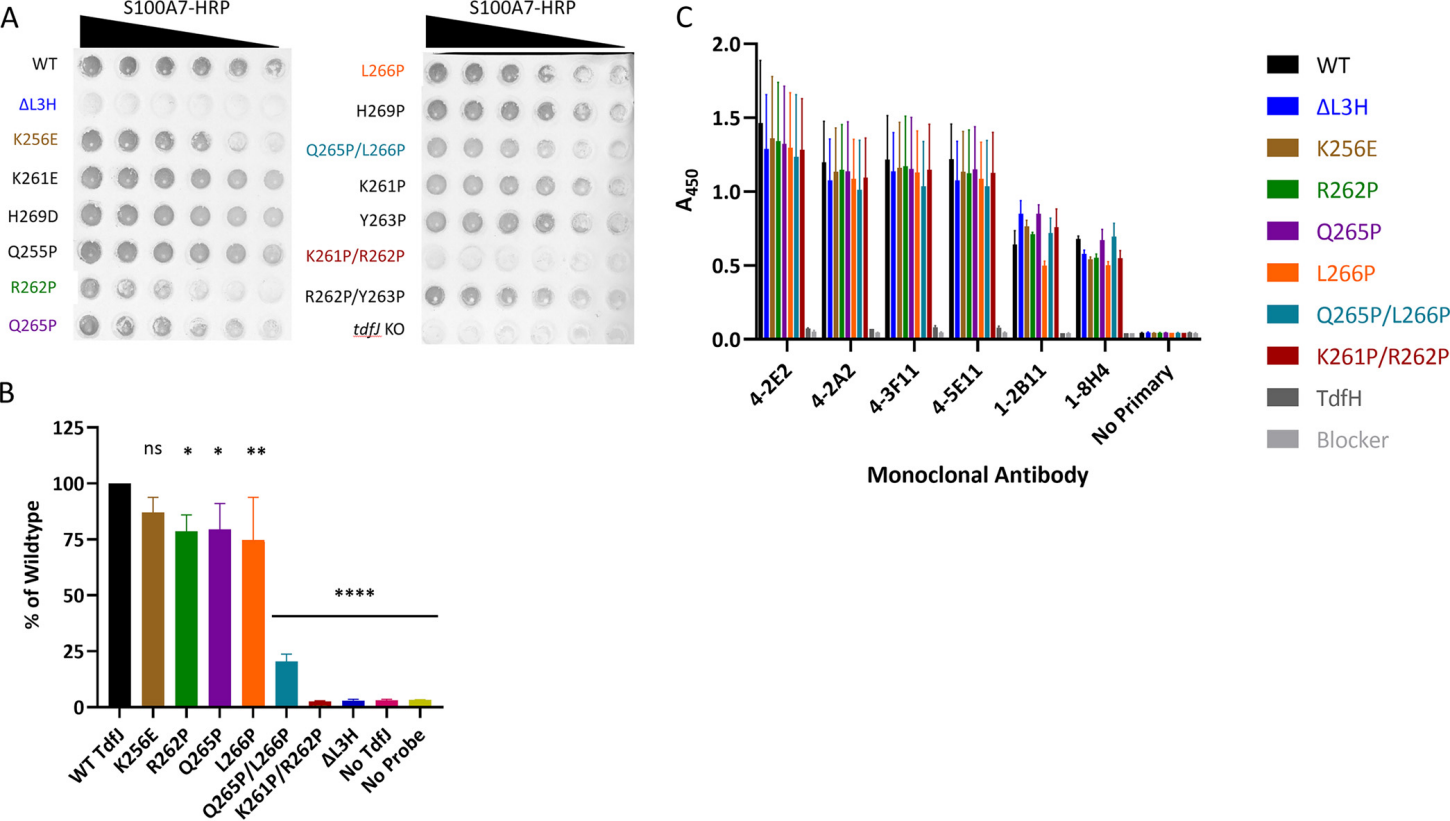
Mutations in the TdfJ loop 3 α-helix can inhibit S100A7 binding. (A) Gonococcal *tdfJ* mutants were grown on GCB agar plates containing 1 mM IPTG and resuspended in PBS to an OD_600_ of 1.0. Suspensions were dotted onto nitrocellulose and allowed to adsorb and dry prior to blocking. The blot was then probed with a dilution series of HRP-labeled S100A7, and the signal was developed with the HRP-reactive CN/DAB substrate. Mutants with appreciable decreases in S100A7 binding are color-coded, and the color convention is maintained for the following figures. The blot is representative of results from 3 experiments. (B) Mutant forms of TdfJ determined to have binding deficiencies based on the results from panel A were purified and used in a quantitative binding assay with S100A7. Purified, His-tagged TdfJ variants were seeded onto an NTA-coated 96-well plate and then blocked with BSA. They were then probed with S100A7-HRP and washed, and the signal was developed with the TMB substrate. The signal was quantified by reading the absorbance at 450 nm, and all samples were calculated as a percentage of the WT. Statistical differences between mutants and the WT were calculated via one-way analysis of variance (ANOVA) (ns, not significant; *, *P* < 0.05; **, *P* < 0.01; ****, *P* < 0.0001). (C) After purification, mutant forms of TdfJ were assessed for stability and folding. Proteins were seeded into a Ni-NTA-coated 96-well plate and subsequently probed with the mAbs described in the legend of Fig. 3. mAb binding was detected via the addition of HRP-labeled anti-mouse IgG, and the signal was quantified by reading the *A*_450_. His-tagged TdfH was added as a control for antibody specificity, and a set of proteins was probed with no primary antibody to assess the background. Statistical differences between the WT and mutants were assessed via two-way ANOVA, and all mutants were statistically indistinguishable from the WT for each mAb. Means and standard deviations from 3 experiments are shown for panels B and C.

To assess TdfJ mutants for their S100A7 binding phenotypes, we first performed a broad screen using whole cells expressing TdfJ. As described above, induced gonococcal cultures were dotted onto nitrocellulose and then probed with a dilution series of horseradish peroxidase (HRP)-labeled S100A7 (S100A7-HRP) (Fig. 4A). We found that certain mutations diminished S100A7 binding to various degrees. The most profound defects were seen in the ΔL3H strain and in a mutant with residues K261 and R262 replaced with prolines (K261P/R262P), both of which generated no detectable S100A7 binding. Minor to moderate binding impacts were also seen in the K256E, R262P, Q265P, L266P, and Q265P/L266P mutants. To investigate these defects further, we purified the above-listed TdfJ proteins and utilized an enzyme-linked immunosorbent assay (ELISA)-based binding assay to assess their defects. His-tagged versions of these proteins were purified, immobilized on a Ni-NTA-coated 96-well plate, and subsequently probed with S100A7-HRP (Fig. 4B). Consistent with the dot blot results, the ΔL3H and K261P/R262P mutants exhibited virtually no S100A7 binding; their *A*_450_ readings were indistinguishable from those of samples with no probe or no target protein added. Additionally, the R262P, Q265P, and L266P mutants demonstrated results consistent with those of the dot blot analyses as well and bound S100A7 at levels that were statistically different from those of the WT but not as profoundly impacted as the previous two. The Q265P/L266P double mutant was somewhat inconsistent between experiments; this mutant appeared roughly similar to the R262P, Q265P, and L266P single mutants in the dot blot but performed significantly worse in the ELISA. The K256E mutant was not statistically distinguishable from WT.

To ensure that the binding defects seen in the ELISA were not due to protein misfolding or instability induced during purification, we utilized the TdfJ-specific mAbs to probe purified TdfJ mutant proteins. The His-tagged TdfJ mutants were seeded onto a Ni-NTA-coated ELISA plate and subsequently probed with the same set of mAbs as the ones that were used in the surface exposure test (Fig. 4C). Notably, all TdfJ variants were recognized by each of the mAbs at levels that were statistically indistinguishable from those of the WT, suggesting that the global fold of the purified proteins mimicked that of their membrane-bound counterparts and that the mutated forms were as well folded as the WT protein. His-tagged TdfH was used as a negative control to demonstrate mAb specificity for TdfJ and was not recognized by the mAbs. The results of these experiments suggest that any differences observed in binding interactions with S100A7 for the mutated forms of TdfJ were not likely to be structural in nature.

**FIG 5:**
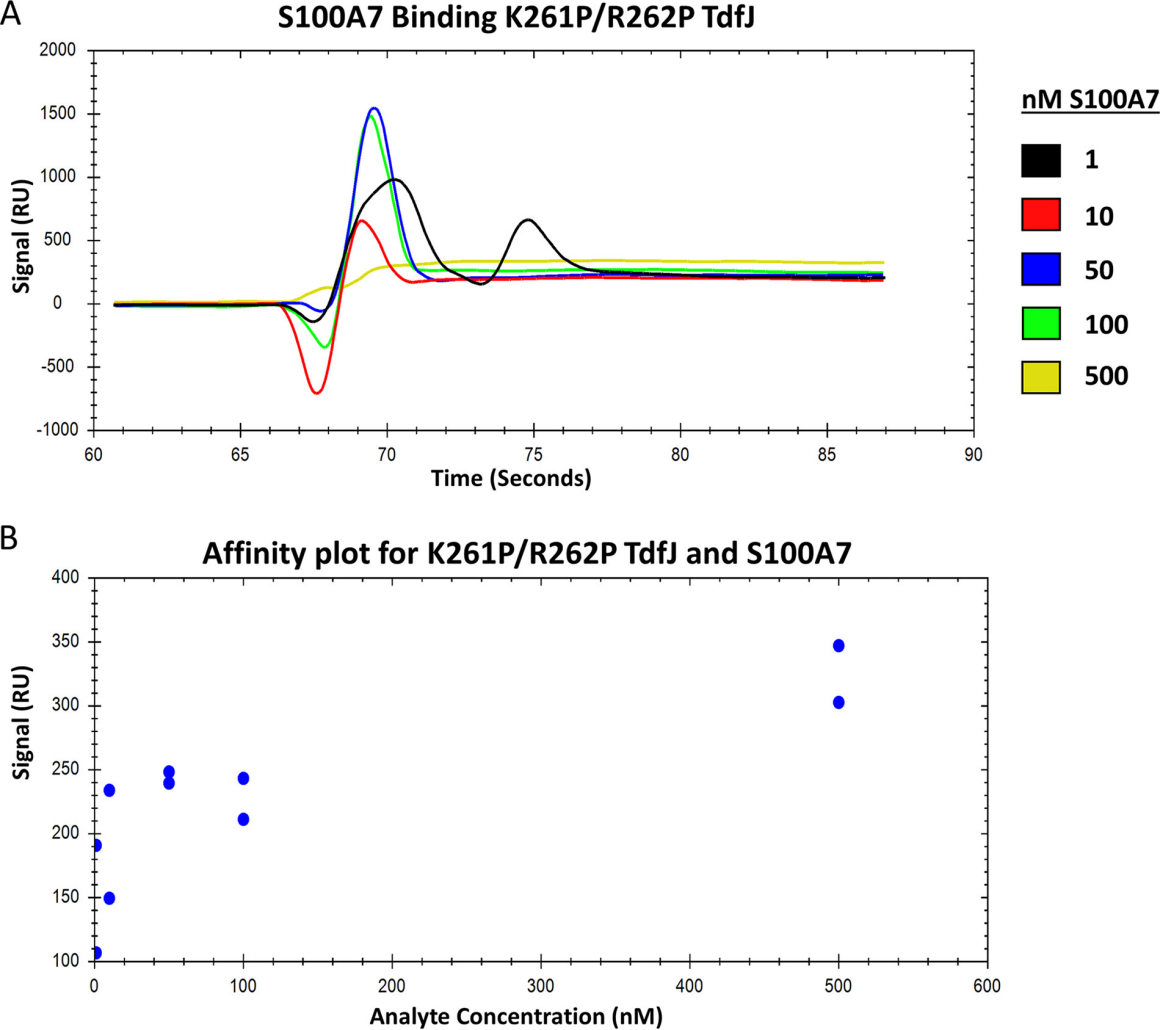
The K261P/R262P TdfJ mutant has a severe S100A7 binding deficiency. His-tagged K261P/R262P TdfJ was immobilized on an NTA-coated SPR sensor chip, and the remaining free NTA sites were blocked with His-tagged streptavidin. S100A7 was then passed over the TdfJ chip to assess binding. (A) An SPR sensorgram showing the baseline, association, and steady-state binding of five concentrations (1, 10, 50, 100, and 500 nM) of S100A7 with K261P/R262P TdfJ. The plot is a representative trace for 2 experiments. (B) Plot showing the steady-state affinity of S100A7 and K261P/R262P TdfJ calculated from two individual traces for each analyte concentration. Affinity could not be calculated using the same model as the one used for the WT in Fig. 1.

We next focused on the mutants that were most defective in our previous binding assays, namely, ΔL3H and K261P/R262P. While both mutants showed similar binding deficiencies, we chose to utilize only K261P/R262P for affinity calculations as the point mutant was a better representative overall for TdfJ than ΔL3H, which contained a large deletion. We performed SPR as described above for the WT protein, using His-tagged K261P/R262P TdfJ as the sensor chip ligand and a 1 to 500 nM analyte series of S100A7 (Fig. 5A). These experiments confirmed the results of our above-described assays, namely, that K261P/R262P was severely defective in its S100A7-binding capability. At the highest concentration of analyte added (500 nM), K261P/R262P generated approximately 325 average RU at equilibrium, only marginally higher than what was seen for 1 nM S100A7 on WT TdfJ and substantially lower than what was seen for the same analyte concentration on the WT (~900 RU). K261P/R262P generated a very slight concentration-dependent response when S100A7 was added, suggesting that its binding signal, while small, was specific to S100A7 and not merely an artifact of analyte injection in general. As with the WT, we attempted an affinity calculation for K261P/R262P. However, fitting the data to the single-site model as used to extract the affinity for the WT protein failed to provide a reasonable fit, and no reliable *K_D_* calculation could be made. While the data could likely be fit to a more complex model with more variables, the inability to fit the data to the simpler model was attributed to the much lower sensitivity of the data as a result of the very small range of the response in the sensorgrams, and we concluded that an alternate model was not merited.

### Gonococci expressing mutated *tdfJ* are defective for S100A7 utilization.

**FIG 6:**
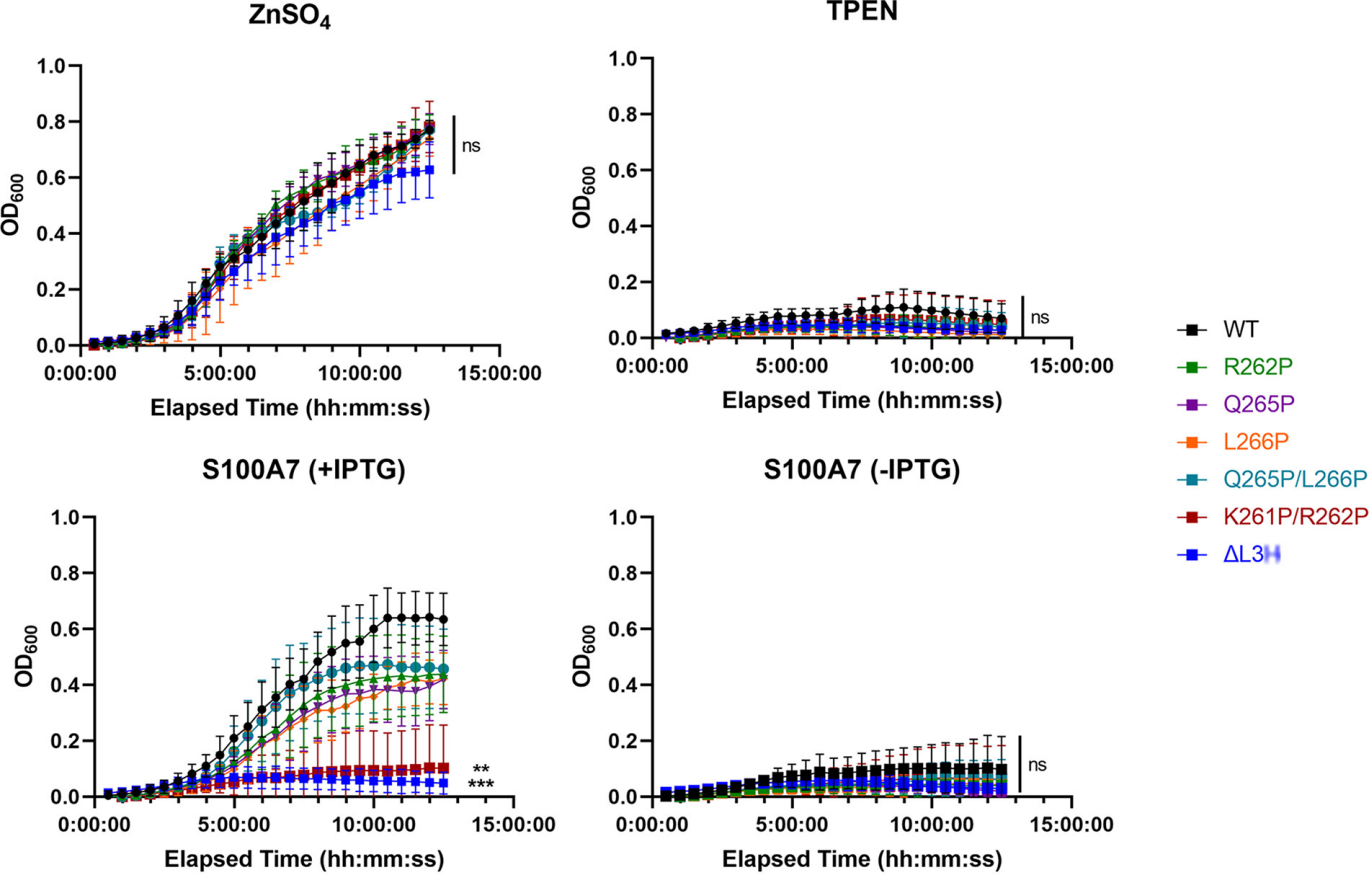
Gonococci expressing mutated *tdfJ* are defective for S100A7 utilization. Gonococcal strains were grown in metal-restricted defined medium until they reached exponential phase to induce zinc starvation. Cells were then back-diluted to an OD_600_ of 0.02 in the same medium and added to 96-well plates supplemented with either ZnSO_4_, TPEN, or TPEN plus zinc-loaded S100A7 with and without IPTG. Cells were grown for 12 h, with the OD_600_ being recorded every 30 min to assess growth. Statistical significance relative to the WT was calculated via two-way repeated-measures ANOVA with Geisser-Greenhouse correction (ns, not significant; **, *P* < 0.01; ***, *P* < 0.001). Means and standard deviations from 3 experiments are shown.

As a consequence of their impaired binding, we next investigated whether other known TdfJ-S100A7 interaction phenotypes were also impacted by mutagenesis. We previously reported that gonococci producing functional TdfJ are able to utilize Zn-loaded S100A7 (Zn-S100A7) as a sole zinc source in metal-depleted medium and that growth in the presence of Zn-S100A7 leads to zinc accumulation within gonococci in a TdfJ-dependent way (18). To determine whether binding deficiencies affected the downstream utilization of Zn-S100A7, we performed growth assays in the same metal-restricted medium as the one used previously, supplemented with Zn-S100A7, and compared the growth of mutant strains to that of the WT (Fig. 6). In this assay, all strains grew equivalently when supplemented with ZnSO_4_ instead of Zn-S100A7, suggesting that baseline growth defects were not present in the TdfJ mutants and that zinc utilization in general was not compromised when a suitable zinc source was present. When samples were fully restricted for zinc by the addition of the zinc-specific chelator *N*,*N*,*N*′,*N*′-tetrakis(2-pyridinylmethyl)-1,2-ethanediamine (TPEN), no growth was observed for any strain. However, when the TPEN-treated samples were also supplemented with Zn-S100A7, gonococci producing TdfJ variants (plus IPTG) exhibited various capacities for growth, consistent with their binding phenotypes. Unsurprisingly, WT TdfJ facilitated the most growth, while the ΔL3H and K261P/R262P samples reached optical densities similar to those of their TPEN-only counterparts, suggesting effectively no capacity for Zn-S100A7 use. Likewise, R262P, Q265P, L266P, and Q265P/L266P fell between the two extremes, consistent with our findings in the binding assays.

**FIG 7:**
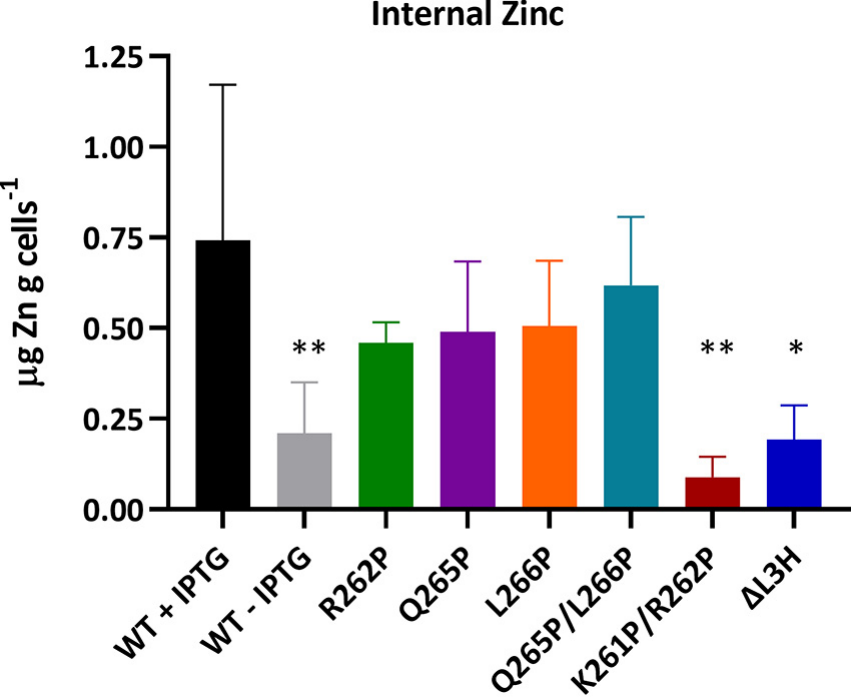
Gonococcal *tdfJ* mutants are defective for zinc acquisition from S100A7. Gonococcal *tdfJ* mutants were grown in metal-restricted medium until exponential phase to induce zinc stress. Cultures were back-diluted in the same medium and supplemented with TPEN and zinc-loaded S100A7, with IPTG added where appropriate. Cultures were grown for an additional 4 h, and cell pellets were then collected via centrifugation. Pellets were washed twice with buffer containing 10 mM HEPES plus 1 mM EDTA and then once more with 10 mM HEPES only. The cell pellets were digested, and the zinc content was assessed via ICP-MS. Statistical differences relative to the WT with IPTG were calculated via one-way ANOVA (*, *P* < 0.05; **, *P* < 0.01). Means and standard deviations are shown for 6 experiments for the two WT samples and 3 experiments for all others.

Finally, we assessed whether TdfJ mutation affected zinc accumulation within the gonococci when Zn-S100A7 was present. We grew cultures in zinc-restricted medium supplemented with Zn-S100A7 and harvested the cell pellets. After the removal of exogenous metals, the cell pellets were analyzed for their zinc content by inductively coupled plasma mass spectrometry (ICP-MS) (Fig. 7). These experiments were consistent with all others and showed that WT TdfJ facilitated the most zinc uptake, while ΔL3H and K261P/R262P allowed the least. All other mutants fell between the extremes as they had done in other assays. Taken together, these data suggest that the α-helical region of TdfJ loop 3, situated between two His-, Asp-, and Glu-rich clusters, plays an essential role in the binding of, and subsequent zinc extraction from, S100A7.

## DISCUSSION

Gonorrhea presents a serious threat to public health, as there is currently no licensed vaccine against the disease and highly drug-resistant isolates of the causative agent, N. gonorrhoeae, continue to emerge. During this pathogen’s life cycle within the human host, it is confronted by host efforts to limit the availability of critical nutrients such as iron and zinc, a concept termed “nutritional immunity,” in order to starve out the infection. In response, the gonococcus deploys eight TdTs to its outer membrane, which serve the critical function of overcoming host nutritional immunity efforts by binding host factors and pirating their metal cargo (reviewed in reference 15). The TbpAB and LbpAB systems facilitate iron acquisition from human transferrin and lactoferrin, respectively (35–37); the HpuAB system allows the gonococcus to extract iron from hemoglobin and hemoglobin-haptoglobin complexes (38, 39); FetA scavenges xenosiderophores from other bacterial species and coopts them for gonococcal use (40); TdfH facilitates zinc acquisition from the innate immunity protein calprotectin (30); and two other TdTs, TdfF and TdfG, do not have a known ligand, but both are repressed by iron, suggesting a role in iron uptake (41). Finally, TdfJ, the topic of this report, is responsible for gonococcal zinc piracy from S100A7 (18).

In this study, we quantified the binding interaction between TdfJ and S100A7 and found a high affinity, with a *K_D_* of 41 nM. This is consistent with other interactions between gonococcal TdTs and their host ligands. For example, TdfH binds calprotectin with nanomolar affinity (42), and a gonococcal strain producing only TbpA and no TbpB binds transferrin with a *K_D_* of approximately 10 nM (43). Additionally, the lipoprotein component of the lactoferrin-iron uptake system, LbpB, binds lactoferrin with a *K_D_* of 140 nM (44). Such high-affinity interactions are not surprising, as the more efficient uptake of iron and zinc during infection would hypothetically offer an evolutionary advantage to the gonococcus. However, it is noteworthy that such strength of interaction may not be entirely necessary, as gonococci seem likely to encounter an abundance of calprotectin and S100A7 during infection. N. gonorrhoeae characteristically stimulates a local influx of neutrophils during infection (45, 46), and calprotectin is abundant within the neutrophil cytosol (47). Similarly, S100A7 is upregulated in response to inflammation and is known to be present in the female genital tract (21), suggesting that it too should colocalize with invading gonococci.

As mentioned above, the crystal structure of meningococcal ZnuD served as the roadmap for our mutagenesis studies, and we focused our efforts on an α-helix motif situated between two zinc-sensing clusters in extracellular loop 3. As discussed previously (25), this region undergoes considerable remodeling in a substrate-dependent way, with the presence of both zinc and cadmium causing the exposed α-helix to collapse into β-strands, presenting a buried, high-affinity zinc site. Because of the implied importance of this region to zinc uptake, we hypothesized that it may be involved in binding to and/or piracy from S100A7. We primarily pursued two routes for mutagenesis. First, we generated a 15-residue deletion in loop 3 between the zinc-sensitive clusters where the α-helix is found. This mutation effectively served as a proof of concept for targeting this region, as any defects should have been apparent from such a significant change. Second, because of the unique architecture of ZnuD regarding its loop-remodeling capabilities and subsequent zinc import, we sought mutations that would cause a disruption to this specific TdfJ motif with a minimal impact on the rest of the protein. To this end, we selected proline substitution, as this cyclic amino acid has high conformational rigidity and is known to disrupt helical secondary structure (48). Mutations of a few charged residues were also designed, with the goal of disrupting electrostatic contributions to the binding of S100A7 and/or perturbing metal coordination in one or both proteins. Interestingly, mutations within the α-helix generated various levels of binding deficiency, with severe defects seen upon proline insertion at residues K261 and R262, which are located centrally within the helix.

The importance of an α-helix in a TdT extracellular loop is not unique to TdfJ. The crystal structure of meningococcal TbpA in complex with transferrin shows such a helix finger in proximity to the transferrin C-lobe, where it is thought to disrupt iron coordination (49). As mentioned above, Cash et al. (29) mutagenized this region and found that certain mutations diminished TbpA-transferrin interactions in the gonococcus, although a proline insertion was not used. A recent report also posited the importance of the TbpA helix in transferrin binding. Duran and Özbil (50) performed molecular dynamics simulations to interrogate the docking of TbpA and transferrin and found that the TbpA loop 3 helix undergoes structural rearrangement upon transferrin binding. Those authors hypothesized that this dynamic allows K359 of TbpA to interact with D634 of transferrin, ultimately resulting in a charge repulsion between transferrin residues K534 and R632, thus opening the binding cleft to free the iron atom. Such a mechanism may be possible for TdfJ and S100A7 as well, although it has not yet been tested. It is important to note that the respective helices of these TdTs do not share appreciable sequence similarity (TbpA, K^351^AVFDANKKQA^361^; TdfJ, Q^255^KSLINKRYLQLYPH^269^), suggesting that the helical structure itself is the predominant conserved property, but further investigation of other TdT-ligand pairs would be needed to validate this.

The TdTs have received considerable interest as vaccine targets for gonorrhea, and TdfJ/ZnuD is no exception to this (51). The TdTs are well conserved across gonococcal isolates and show a limited propensity for antigenic variation, which has stymied efforts targeting many other surface structures. In addition to their conserved nature, the importance of the TdTs to gonococcal infection cannot be overlooked. An N. gonorrhoeae strain, FA1090, with an inactivated transferrin receptor system is unable to cause infection in human males (52); TdfH allows gonococci to survive within neutrophil extracellular traps (NETs) (30); TdfF is required for gonococci to replicate within cervical epithelial cells (53); and ZnuD contributes to meningococcal interactions with epithelial cells, and *znuD* mutants are defective for dissemination in a mouse model (25, 54). Such key virulence factors are highly promising as vaccine and/or therapeutic targets, and mutagenesis of such targets has received attention with regard to generating a robust, protective immune response. A few recent studies have focused on identifying mutated forms of bacterial structures that are unable to bind the host ligands that are recognized by their wild-type counterparts, acting on the hypothesis that a bound host factor may dampen the immune recognition of said bacterial target. Such studies by Frandoloso et al. and Martínez-Martínez et al. (55, 56) mutagenized the TbpB protein found in the pig pathogen Haemophilus parasuis and demonstrated that a transferrin-binding-defective mutant, which retained a wild-type-like conformation, conferred superior protection against bacterial challenge compared to WT TbpB and also elicited more robust B- and T-cell responses. Beernink et al. similarly generated nonbinding mutants of meningococcal factor H-binding protein (fHbp) and found that upon immunizing factor H transgenic mice, a nonbinding mutant elicited more bactericidal antibodies and more factor H-blocking antibodies than what was seen for WT fHbp (57). However, the mutagenesis paradigm does not appear to be universally true, as previous fHbp mutagenesis efforts by Beernink et al. showed an impaired immune response and compromised a key immunogenic region of fHbp (58). To date, no studies have been published that have investigated whether a nonbinding version of a TdT may confer superior protection and/or immune stimulation, so one must be cautious about discussing their potential superiority as antigens. Nevertheless, a future study utilizing such TdT mutants, including the one(s) described in this report, may be an interesting path moving forward.

In summary, here, we report for the first time the binding interaction for gonococcal TdfJ and its human ligand S100A7. We also identified several mutations in TdfJ loop 3 that alter S100A7 binding and subsequent zinc extraction. Because the TdTs continue to feature as promising targets for vaccine and therapeutic development to combat gonococcal disease, an intimate understanding of their virulence mechanisms is of paramount importance, and similar characterization of the other TdT-ligand pairs will hopefully follow.

## MATERIALS AND METHODS

### Gonococcal growth conditions.

Strains of N. gonorrhoeae were maintained on GC medium base (Difco) agar with Kellogg’s supplement I (59) and 12.4 μM Fe(NO_3_)_3_ (GCB agar plates) at 36°C with 5% CO_2_. Where appropriate, GCB plates were supplemented with 1 mM IPTG. For metal-restricted growth, gonococcal cells were inoculated to a final optical density at 600 nm (OD_600_) of 0.09 in 5 mL of defined medium that was pretreated with Chelex-100 resin (Bio-Rad) (Chelex-treated defined medium [CDM]) in beveled sidearm flasks. Cultures were grown at 36°C with 5% CO_2_, with shaking at 225 rpm.

### Gonococcal mutant construction.

All strains and plasmids utilized in this study are summarized in Table 1. Primer sequences and plasmid maps are available upon request. Restriction endonucleases were acquired from New England BioLabs (NEB). To construct the ectopic *tdfJ* mutants, mutated *tdfJ* gene sequences were submitted to either Bio Basic Inc. or Genewiz Inc. for *de novo* synthesis and subsequently cloned into pVCU234 between the XmaI and XhoI sites. The XmaI site was reconstructed during this cloning step, leaving 6 nucleotides between the plasmid’s ribosome-binding site and the *tdfJ* start codon. For gonococcal transformations, plasmids were first linearized with PciI and then used to transform a piliated population of gonococcal strain MCV928 (31). Transformants were recovered on GCB plates supplemented with 1 μg/mL chloramphenicol, and the presence of the *tdfJ* gene(s) was verified by PCR.

**TABLE 1:** Strains and plasmids used in this study

Strain or plasmid	Genotype or purpose	Source or reference
Strains		
E. coli		
TOP10	F^−^ *mcrA* Δ(*mrr-hsdRMS-mcrBC*) ϕ80*lacZ*ΔM15 Δ*lacX74 nupG recA1 araD139* Δ(*ara-leu*)*7697 galE15 galK16 rpsL*(Str^r^) *endA1* λ^−^	Invitrogen
DH5α	F^−^ *endA1 glnV44 thi-1 recA1 relA1 gyrA96 deoR nupG purB20* ϕ80d*lacZ*ΔM15 Δ(*lacZYA-argF*)*U169 hsdR17*(r_K_^−^ m_K_^+^) λ^−^	NEB
OverExpress C41(DE3)	F^−^ *ompT gal dcm hsdS*_B_(r_B_^−^ m_B_^−^)(DE3)	Lucigen
Gonococcal		
FA19	Wild type	33
MCV928	FA19 *tdfJ*::Ω	31
RSC010	MCV928 + ectopic WT *tdfJ*	This study
RSC011	MCV928 + ectopic L3H deletion *tdfJ*	This study
RSC002	MCV928 + K256E mutant *tdfJ*	This study
RSC003	MCV928 + K261E mutant *tdfJ*	This study
RSC004	MCV928 + H269D mutant *tdfJ*	This study
RSC005	MCV928 + Q255P mutant *tdfJ*	This study
RSC006	MCV928 + R262P mutant *tdfJ*	This study
RSC007	MCV928 + Q265P mutant *tdfJ*	This study
RSC008	MCV928 + L266P mutant *tdfJ*	This study
RSC009	MCV928 + H269P mutant *tdfJ*	This study
RSC012	MCV928 + Q265P/L266P mutant *tdfJ*	This study
RSC013	MCV928 + K261P mutant *tdfJ*	This study
RSC014	MCV928 + Y263P mutant *tdfJ*	This study
RSC015	MCV928 + K261P/R262P mutant *tdfJ*	This study
RSC016	MCV928 + R262P/Y263P mutant *tdfJ*	This study
Plasmids		
pET-22b-pso	pET-22b containing the S100A7 coding sequence	61
pVCU234	pKH37 + ribosome-binding site	32
pVCU554	pVCU234 + WT *tdfJ*	18
pGSU021	pVCU234 + L3H deletion *tdfJ*	This study
pGSU024	pVCU234 + K261E *tdfJ*	This study
pGSU025	pVCU234 + K256E *tdfJ*	This study
pGSU026	pVCU234 + H269D *tdfJ*	This study
pGSU027	pVCU234 + Q255P *tdfJ*	This study
pGSU028	pVCU234 + R262P *tdfJ*	This study
pGSU029	pVCU234 + Q265P *tdfJ*	This study
pGSU030	pVCU234 + L266P *tdfJ*	This study
pGSU031	pVCU234 + H269P *tdfJ*	This study
pGSU324	pVCU234 + Q256P/L266P *tdfJ*	This study
pGSU325	pVCU234 + K261P *tdfJ*	This study
pGSU326	pVCU234 + Y263P *tdfJ*	This study
pGSU327	pVCU234 + K261P/R262P *tdfJ*	This study
pGSU328	pVCU234 + R262P/Y263P *tdfJ*	This study
pET-20bHT	His-tagged expression vector with the *pelB* signal sequence	60
pGSU023	pET-20bHT + mature WT *tdfJ*	This study
pGSU318	pET-20bHT + mature L3H deletion *tdfJ*	This study
pGSU319	pET-20bHT + mature K256E *tdfJ*	This study
pGSU320	pET-20bHT + mature R262P *tdfJ*	This study
pGSU321	pET-20bHT + mature Q265P *tdfJ*	This study
pGSU322	pET-20bHT + mature L266P *tdfJ*	This study
pGSU329	pET-20bHT + mature Q265P/L266P *tdfJ*	This study
pGSU331	pET-20bHT + mature K261P/R262P *tdfJ*	This study

### Expression plasmid construction.

To generate expression systems for recombinant TdfJ, the mature coding region of WT and mutant *tdfJ* was amplified from the complementation plasmids by PCR, and fragments were subsequently cloned via the In-Fusion system (Clontech) into pET-20bHT (60) between the NcoI and XhoI sites. pET-20bHT contains a *pelB* signal sequence followed by a 10× N-terminal His tag and a tobacco etch virus (TEV) cleavage site. Clones were used to transform Escherichia coli cells, which were recovered on 100 μg/mL carbenicillin and verified by PCR.

### Recombinant protein purification.

Human S100A7 was produced from a pET-22b expression vector provided by Joachim Grötzinger (pET-22b-pso) using the protocol described previously by Grötzinger and coworkers (61). His-tagged TdfH was purified as described previously, with the tag cleavage step omitted (42). Wild-type and mutant TdfJ were produced from E. coli C41(DE3). For purification, a starter culture of the appropriate plasmid was prepared in Luria-Bertani (LB) medium supplemented with 100 μg/mL carbenicillin and subcultured into Terrific broth (TB) with the same antibiotic. Expression cultures were grown without the inducer for approximately 48 h at 20°C, and cell pellets were harvested by centrifugation (10,000 × *g* for 1 h at 4°C). Cell pellets were resuspended in lysis buffer (20 mM HEPES [pH 8.0], 250 mM NaCl, 100 μg/mL lysozyme, 1 mM phenylmethylsulfonyl fluoride [PMSF]) using a Dounce homogenizer, with 10 mL of buffer used per g of cell paste. Cell suspensions were then mechanically lysed via two passages through an Emulsiflex C3 instrument (Avestin Inc.) with a homogenizing pressure of ~17,500 lb/in^2^. Insoluble material was removed via centrifugation (30,000 × *g* for 30 min at 4°C), and the supernatant was collected and mixed with 1% Triton X-100 for 1 h at room temperature. The supernatant was then centrifuged at 160,000 × *g* for 1 h to pellet the membranes, and the remaining supernatant was discarded. The membranes were resuspended in a minimal volume of membrane buffer (20 mM HEPES [pH 8.0], 250 mM NaCl, 10% Elugent, 1 mM PMSF) and allowed to mix at 4°C overnight. Undissolved membranes were then pelleted via centrifugation (12,500 × *g* for 20 min), and the remaining solubilized material was mixed with Ni-NTA resin for 2 to 3 h at 4°C. The resin was collected in a chromatography column and then washed with 10 column volumes (CV) each of wash buffer (20 mM HEPES [pH 8.0], 150 mM NaCl, 0.25% Elugent, 1 mM PMSF) containing 0, 50, 100, 200, and 300 mM imidazole. TdfJ eluted cleanly at 300 mM. The eluted protein was then dialyzed overnight at 4°C into phosphate-buffered saline (PBS) (pH 8.0) plus 0.25% Elugent and, if necessary, concentrated using a 50,000-Da-cutoff centrifugal filter. Protein was then aliquoted and flash-frozen at −80°C.

### Surface plasmon resonance.

All SPR experiments were performed using an OpenSPR XT instrument (Nicoya), and trace analysis was conducted using TraceDrawer (Ridgeview). All reagents were prepared according to the manufacturer’s instructions, using degassed buffers. TdfJ and S100A7 were buffer exchanged into degassed PBS (pH 8.0) prior to experiments, and all dilutions were performed using the same buffer. His-tagged streptavidin was reconstituted in degassed distilled water (dH_2_O) prior to use. For binding experiments, NTA-coated sensor chips were first cleaned via successive injections of 10 mM HCl (150 μL/min) and 350 mM EDTA (100 μL/min), followed by surface activation with an injection of 40 mM NiCl_2_ (20 μL/min). For ligand immobilization, WT or K261P/R262P TdfJ containing a 10× N-terminal His tag was injected into flow channel 2 at a concentration of 89 μg/mL (20 μL/min), and the remaining NTA groups were blocked via the addition of 50 μg/mL His-tagged streptavidin into both channels 1 and 2 (20 μL/min), allowing channel 1 to serve as a reference for nonspecific interactions. S100A7 was injected (20 μL/min) into both channels in successive steps using the following concentrations: 1, 10, 50, 100, and 500 nM. No chip regeneration step was found to be necessary. Sensorgrams were exported to TraceDrawer, where readouts for each concentration were aligned and bubble peaks were removed. For affinity analysis, sensorgrams from multiple, independent experiments were combined, aligned, and used to generate a single binding curve interval, which was then analyzed using a single-site fit model and the EC_50_ analysis setting. In total, 13 data points were used in the WT affinity model, and 10 were used in the attempted fit of the K261P/R262P mutant.

### Whole-cell dot blots.

Gonococcal strains were grown on GCB agar plates with or without 1 mM IPTG before being resuspended in PBS. Cell suspensions were standardized to an OD_600_ of 1.0 before being dotted onto nitrocellulose in a dot blotter, cells were then allowed to adsorb, and the blots were left to dry. The dried blots were blocked with either 5% (wt/vol) bovine serum albumin (BSA) or 5% (wt/vol) nonfat dry milk dissolved in Tris-buffered saline plus 0.05% Tween 20 (TBST). For S100A7 binding assays, blots were returned to the dot blotter and probed with horseradish peroxidase (HRP)-labeled S100A7 (S100A7-HRP) dissolved in blocker at the following concentrations: 0.4, 0.2, 0.1, 0.05, 0.025, and 0.0125 μM. After probing for 1 h at room temperature, the probe was removed via a vacuum, and the blot was washed three times for 10 min using TBST. The HRP signal was developed using the 4-Chloro-1-naphthol and 3,3′-Diaminobenzidine mixture (CN/DAB) substrate (Thermo Fisher). For monoclonal antibody probing during mAb characterization, dried and blocked blots were returned to the blotter and probed with a hybridoma supernatant containing the mAbs diluted in blocker, as follows: 1:10 → 1:50 → 1:100 for 4-2E2, 4-2A2, 4-3F11, and 4-5E11 and 1:10 → 1:20 → 1:40 for 1-2B11 and 1-8H4. Probing was performed at 4°C overnight. For mutant surface exposure and folding analyses, dried and blocked blots were probed with 4-2A2 (IgG1; 1:25 in blocker), 4-2E2 (IgG2a; 1:200), 4-3F11 (IgG1; 1:10), 4-5E11 (IgG1; 1:10), 1-2B11 (IgG1; 1:10), and 1-8H4 (IgG2b; 1:5), also at 4°C overnight. Following primary probes, blots were washed three times with TBST and then probed for 1 h at room temperature with either alkaline phosphatase (AP)- or HRP-labeled anti-mouse IgG secondary antibodies (1:3,000 in blocker). Blots were washed again, and the signal was developed with the CN/DAB substrate for HRP and nitroblue tetrazolium (NBT)–5-bromo-4-chloro-3-indolylphosphate (BCIP) for AP.

### Purified protein ELISAs.

For conformational testing with the mAbs, 100 μL of His-tagged TdfJ (WT and mutants) or TdfH was seeded into the wells of a Ni-NTA-coated ELISA plate at 1 μM. Proteins were allowed to bind for 2 h at room temperature or overnight at 4°C. Protein was siphoned off by a vacuum, and wells were blocked with 200 μL of 5% (wt/vol) BSA in TBST for 1 h at room temperature. After blocking, the wells were probed with 100 μL of the mAb (4-2A2, 4-2E2, 4-3F11, 4-5E11, 1-2B11, or 1-8H4) supernatant diluted in blocker (1:100 for all) for 2 h at room temperature. Liquid was again siphoned off, and the wells were washed three times with 200 μL TBST for 10 min each. HRP-labeled anti-mouse IgG was diluted in blocker (1:3,000), and 100 μL was added to the wells for secondary probing for 1 h at room temperature. After siphoning and three more washes, the HRP signal was developed by the addition of 100 μL of the 3,3′,5,5′-Tetramethylbenzidine (TMB) substrate, and the reaction was neutralized with 100 μL of 180 mM H_2_SO_4_. Coloration was quantified by reading the absorbance at 450 nm using a Cytation5 plate reader (BioTek). For S100A7 binding assays, 100 μL of His-tagged TdfJ (WT and mutants) was seeded into the Ni-NTA plates at 1 μM and allowed to bind for 1 h at room temperature. Wells were blocked as described above, 100 μL of 1 nM S100A7-HRP in blocker was then added to the wells, and the wells were probed for 1 h. Liquid was again siphoned away, and the wells were washed three times with 200 μL TBST. The HRP signal was developed as described above, and all *A*_450_ values were standardized as a percentage of wild-type binding.

### Zinc-restricted growth assay.

Gonococcal growth assays under zinc-restricted conditions were performed as previously described (62). In short, gonococci were grown to exponential phase in CDM to induce zinc stress. At this point, cultures were back-diluted to an OD_600_ of 0.02 in the same medium and added to a 96-well plate, where they were supplemented with either 5 μM ZnSO_4_ as a positive control, 5 μM TPEN as a negative control, or 5 μM TPEN plus 5 μM Zn-loaded S100A7 to test S100A7 utilization. A total of 1 mM IPTG was added where appropriate. Cultures were grown for 12 h (36°C with 5% CO_2_ at 225 rpm) in a Cytation5 plate reader (BioTek), with the OD_600_ being measured every 30 min to track growth.

### Zinc internalization assay.

Gonococcal cultures were grown as described above in CDM until exponential phase. At this point, cultures were back-diluted to one-half their original density and supplemented with 1 μM Zn-loaded S100A7 and 1 mM IPTG where appropriate. Cultures were grown for 4 h before cell pellets were harvested by centrifugation (10,000 × *g* for 10 min). Pellets were washed twice with buffer containing 10 mM HEPES plus 1 mM EDTA and then once more with 10 mM HEPES only. Cell pellets were frozen at −20°C before being sent to the Plasma Chemistry Laboratory at the University of Georgia Center for Applied Isotope Studies for ICP-MS analysis. Data are reported as micrograms of zinc per gram of cell pellet.

### Western blotting.

Whole-cell lysates of gonococci were harvested by pelleting cultures at a standardized optical density (100 Klett units in 1 mL of culture) and resuspending cells in 2× Laemmli solubilizing buffer before storage at −20°C. Immediately preceding use, samples were thawed, mixed with 5% β-mercaptoethanol, and boiled for 5 min. Protein samples were separated on a precast 4-to-20% gradient polyacrylamide gel before transfer to nitrocellulose. The blots were stained with Ponceau S to verify equal protein sample loading. To detect TdfJ, the blots were first blocked in 5% (wt/vol) nonfat dry milk dissolved in TBST. The blots were then probed with either TdfJ peptide-specific guinea pig polyclonal antiserum (1:200 in blocker) or the mAb hybridoma supernatant (1:100 in blocker) for 2 h at room temperature. The generation of the TdfJ peptide antiserum was described previously (63). The blots were washed three times with TBST and then probed with either HRP-conjugated anti-guinea pig IgG or HRP-conjugated anti-mouse IgG secondary antibodies (1:3,000 in blocker) for 1 h. The blots were then washed again, developed using the SuperSignal West Femto extended-duration substrate (Thermo Fisher), and imaged on a Bio-Rad ChemiDoc gel imaging system using 4-by-4 auto-ECL detection.
